# Ultrasonic Assessment of the Medial Temporal Lobe Tissue Displacements in Alzheimer’s Disease

**DOI:** 10.3390/diagnostics10070452

**Published:** 2020-07-03

**Authors:** Mindaugas Baranauskas, Rytis Jurkonis, Arūnas Lukoševičius, Monika Makūnaitė, Vaidas Matijošaitis, Rymantė Gleiznienė, Daiva Rastenytė

**Affiliations:** 1 Biomedical Engineering Institute, Kaunas University of Technology, K. Baršausko g. 59-454, LT-44029 Kaunas, Lithuania; rytis.jurkonis@ktu.lt (R.J.); arunas.lukosevicius@ktu.lt (A.L.); monika.makunaite@ktu.edu (M.M.); 2 Lithuanian University of Health Sciences, A. Mickevičiaus g. 9, LT-44307 Kaunas, Lithuania; vaidas.matijosaitis@lsmuni.lt (V.M.); rymante.gleizniene@lsmuni.lt (R.G.); daiva.rastenyte@lsmuni.lt (D.R.)

**Keywords:** Alzheimer’s disease, brain pulsation, strain, sonography, radiofrequency ultrasound, diagnostic

## Abstract

We aim to estimate brain tissue displacements in the medial temporal lobe (MTL) using backscattered ultrasound radiofrequency (US RF) signals, and to assess the diagnostic ability of brain tissue displacement parameters for the differentiation of patients with Alzheimer’s disease (AD) from healthy controls (HC). Standard neuropsychological evaluation and transcranial sonography (TCS) for endogenous brain tissue motion data collection are performed for 20 patients with AD and for 20 age- and sex-matched HC in a prospective manner. Essential modifications of our previous method in US waveform parametrization, raising the confidence of micrometer-range displacement signals in the presence of noise, are done. Four logistic regression models are constructed, and receiver operating characteristic (ROC) curve analyses are applied. All models have cut-offs from 61.0 to 68.5% and separate AD patients from HC with a sensitivity of 89.5% and a specificity of 100%. The area under a ROC curve of predicted probability in all models is excellent (from 95.2 to 95.7%). According to our models, AD patients can be differentiated from HC by a sharper morphology of some individual MTL spatial point displacements (i.e., by spreading the spectrum of displacements to the high-end frequencies with higher variability across spatial points within a region), by lower displacement amplitude differences between adjacent spatial points (i.e., lower strain), and by a higher interaction of these attributes.

## 1. Introduction

Alzheimer’s disease (AD) is one of the most common neurodegenerative disorders in Europe and in the world [1]. The main pathologic characteristic of AD is cerebral beta-amyloidosis and neurodegeneration hallmarked by amyloid plaque and neuronal tangles upon microscopic examination. Discovery of AD biomarkers, such as cerebrospinal fluid (CSF) amyloid β1-42, CSF tau, or imaging ones (e.g., amyloid positron emission tomography (PET) imaging, fluorodeoxyglucose (^18^F-FDG) PET imaging, volumetric magnetic resonance imaging (MRI)), were enormous during the last decades [2,3]. Despite that, there is still a need for an easily accessible, reliable, safe, and affordable diagnostic tool.

Transcranial sonography (TCS) is a non-invasive, relatively cheap, and portable ultrasound (US) method which allows to us investigate and evaluate brain tissue echogenicity. TCS has been proven to be a reliable diagnostic tool in the differential diagnosis of Parkinsonian syndromes [4,5], and a good method to measure focal and global brain atrophy [6]. Studies have shown that atrophy of the medial temporal lobe (MTL) is a reliable imaging marker that can help in differentiating AD patients from relatively healthy ones [7,8,9,10]. Seen in a recent study by Yilmaz et al., [11], the US technique of the MTL assessment was presented as a possible additional method supporting the diagnosis of AD, which could be comparable to the results of an MRI [2]. These mentioned TCS techniques are based only on imaging of echo intensity in US B mode, allowing them to assess brain structures at a single moment.

TSC could be used not just in conventional B mode, however. It is possible to track the brain tissue displacements at the scale of micrometers caused by the endogenous quasi-periodic pulsatility in the intracranial basin. These displacements can be evaluated while analyzing raw US radiofrequency (RF) signal changes of the same tissue region over time [12]. Brain tissue biomechanics and endogenous tissue displacement characteristics are likely to be affected by developing pathology and correlated with tissue stiffness [13]. Small tissue deformations caused by endogenous intracranial pulsatility were used for strain elastography derived from US data as well [14]. Ultrasound elastography in cerebral applications still are rarely researched [15,16]. Elastographic techniques can be broadly classified based on the mechanical stimuli [17]. Our approach is based on physiological excitation. Implementation of raw US RF signals to track tissue micromovements allows us to derive new parameters of repeatable displacement waveforms. Amplitudes of displacements can be used for the strain estimates in intracranial structures [14,18]. The waveform of displacement can be characterized by amplitude [19], by root mean square [20], or by the parameters of frequency spectra [21,22,23,24]. The frequency spectrum of signals obtained using different monitoring technologies—invasive CSF pressure [25], intracranial pressure [26,27,28,29,30,31], functional magnetic resonance imaging [32,33], transcranial color Doppler imaging [23]—reveals a wide frequency bandwidth of intracranial dynamics. However, analysis of endogenous tissue displacement spectra, registered locally in brain tissue, is still lacking, although it is potentially important in the context of developing pathology, for instance, neurodegeneration.

Therefore, comprehensive analysis of US RF signals obtained from the pulsating brain tissue and calculation of new parameters of brain tissue displacements could help to develop more accurate US RF-based diagnostic methods. During our previous study, while developing a US RF method of displacement quantification [20], we presented a possibility to apply this method for one patient with Alzheimer’s disease (AD) however, to our knowledge, the tissue displacement method was not previously tested for AD diagnostics in a sample of patients.

Since medial temporal lobe atrophy is considered to be one of the hallmarks of AD [34], we aim to estimate brain tissue displacements in that particular region of the brain using backscattered US RF signals. Brain tissue displacement parameters obtained from US RF signals are used to assess the diagnostic ability to differentiate patients with AD from healthy controls. To improve the diagnostic value of the method, we also present essential modifications of our previous method [20] in US waveform parametrization, increasing the statistical certainty of micrometer-range displacement signals in the presence of noise, and analysis of regression models.

## 2. Materials and Methods

The study design and consent procedures were approved by the Ethics Committee for Biomedical Research at the Lithuanian University of Health Sciences (decision on 19th December 2017, No. BE-2-728), Kaunas, Lithuania. All participants gave signed informed consent prior to inclusion in the study. All procedures were in accordance with the Declaration of Helsinki.

### 2.1. Patients and Control Group

Twenty AD patients were prospectively selected from both outpatient and inpatient units of the Department of Neurology at the Hospital of Lithuanian University of Health Sciences Kaunas Clinics (Kaunas, Lithuania) from March 2018 until March 2019. Neurologists who investigated the patients for a possible cognitive disorder performed an initial selection of potential study subjects. Patients were included in the study if they: (1) were diagnosed with possible sporadic Alzheimer’s disease based on the National Institute of Neurological and Communicative Disorders and Stroke (NINCDS) and the Alzheimer’s Disease and Related Disorders Association (ADRDA) criteria [35]; (2) provided written consent; (3) had satisfactory acoustic window properties on at least one side for TCS; (4) were 18 years or older; (5) were eligible for MRI. Patients were excluded from the study if they: (1) refused to have the examination; (2) were younger than 18 years old; (3) had bilateral temporal acoustic bone insufficiency; (4) their diagnosis was uncertain; (5) had a major somatic disease (decompensated heart failure, terminal renal or hepatic dysfunction, active cancer, had diagnosed hemodynamically significant intracranial/extracranial artery stenosis or thrombosis); (6) had a severe mental disorder (psychotic type, severe depression); (7) were on continuous or prolonged use of medications affecting cognitive functions; (8) had a prominent neurological deficit (severe visual disturbance, aphasia, severe paresis, ataxia, evident extrapyramidal signs).

Twenty age- and sex-matched control subjects were recruited from healthy relatives of the patients, provided they did not have any major somatic illness as listed above, cognitive impairment or any structurally abnormal findings in brain MRI and were not under investigation or treatment for any neurodegenerative disease. All participants were Caucasians and most of them were of Lithuanian origin.

All participants completed a questionnaire on general demographic information and risk factors. Education was evaluated by the duration of formal education in years. Family history was considered positive if there was at least one known AD case among first- or second-degree relatives. Both patients and control subjects underwent a standard neurological examination and a standard neuropsychological evaluation by means of a Mini-Mental State Examination (MMSE) test. Each patient with AD was consulted by a psychiatrist and a psychologist to rule out pseudodementia.

### 2.2. Transcranial Sonography (TCS)

TCS imaging for endogenous brain tissue motion data collection was performed using a research-dedicated ultrasound scanner, Ultrasonix Sonix Touch (Analogic Ultrasound, Canada, 2013), equipped with a phased array sector probe (SA 4–2, 64 acoustic elements). The main parameters of ultrasonic scanning and radiofrequency (RF) signal digitization were as follows: sampling frequency fs = 40 MHz, analog-to-digital converter resolution 16 bits, number of post-beam formed scanning lines—131, angle of phased array transducer sector—60°, scanning depth—11 cm, frequency of ultrasound waves 2.5 MHz, frame rate—45 Hz, transmit focal single depth—7 cm. The sequences for recording B-mode scans (259 frames in total) and raw B scan forming RF signals were acquired and stored for off-line analysis.

All US scans were performed by an experienced neurosonologist (Vaidas Matijošaitis) with 13 years of experience in intracranial vascular and structural ultrasound. All study subjects were asked to stay calm and relaxed in a supine position for 5 min and remain motionless and speechless during the examination. First scans were made in a mid-brain scanning plane (axial) in B-mode through a right temporal bone window. The transducer was then rotated by 90° around its longitudinal axis, and the MTL was identified as a brain structure limited by a choroidal fissure in a coronal plane. Position of the transducer was fixed by closing the dedicated spherical bearing of the bracket during insonation of the MTL. The investigator had no contact with the patient, US transducer or the patient’s bed during US RF signal recording. Subsequent to recording the sequence of B-mode scan images, the MTL was marked as a region of interest (ROI) for further investigation, and 6-s length US RF signal sequences were stored in the computer memory. All procedures described above were repeated while making scans through a left temporal bone window. Thus, two ROIs—one from the left and another from the right side of the head—were investigated for each study subject, and the acquired signals were analyzed separately.

### 2.3. Quantification of Brain Tissue Displacements

Quantification of intracranial displacements had five steps of processing:

- RF signal processing to obtain spatial point displacements in ROI
- displacement signal post-processing
- selection of individual confident spatial points
- displacement quantification of individual points
- quantification distribution of displacement of individual points

The classical 1D cross-correlation was used for RF signal processing [19,36,37] to obtain displacements along the scanning line. The sensitivity of the displacement estimator was the same as our previous study [20] but, in the present study, several essential modifications of displacement signals post-processing were introduced. We have excluded averaging of the signal inside the ROI and added two steps before high-pass filtering:

- The accumulated displacement signal was calculated by respecting the change of coordinates of moved points (i.e., the Lagrangian coordinate system [38]) rather than summing inter-frame displacements at particular coordinates (i.e., the Eulerian coordinate system).
- The accumulated displacement signal then was corrected by subtracting the median for each scanning frame’s line separately to contrast micromovements within the ROI; this also was to remove a common movement of the entire ROI caused by possible movement of the transducer in respect to the head of the patient.

Assessment of displacement confidence was modified as well:

- The averaged signal of all spatial points with a dominant frequency (FreqD) in displacement low-end spectra (from 0.67 to 2.00 Hz interval) was used to determine global time windows (that were used for waveform analysis in later steps) in our previous study. The frequency interval of 0.67–2.00 Hz was determined empirically, as within this interval we found the spectrum peak corresponding to the repeatable waveform of displacements in US RF signals (supposedly caused by heart beats) from all subjects. Seen in a current study, median correction was applied, in this case only points with motion pattern in the same direction were used to avoid annihilation, by averaging movements to the opposite directions.
- Global time windows of 600 ms were determined by a normalized correlation coefficient ≥ 0.9 (rather than ≥ 0.8 in our previous study) between an auto-selected pattern and segments of an averaged signal of spatial points mentioned above. When the averaged signal had less than three consecutive similar (not well correlated) segments (including pattern segment), displacement data were rejected from further analysis.
- Considering every spatial point separately (also for the points not included into signal averaging), the reliability of its accumulated displacement signal was evaluated by correlations of signal segments based on identified global time windows. Previously, a confident spatial point was needed to positively correlate every pair of such segments between each other. During a current study, reliability was evaluated by signal segment correlations with the average of these segments of this particular spatial point. This improvement allows us to not reject a spatial point in case only one correlation between its movement segments is not sufficient, as well as speed calculations.

The accumulated waveforms of displacement signals were quantified using three groups of the displacement parameters for each individual confident spatial point (i.e., for the points with a significantly repeatable movement):

1. Displacement amplitude parameters:
  - Root mean square (RMS)—represents displacement intensity and is calculated as the square root of the mean over an entire recorded time (6-s) of the displacement signal square. This metric of amplitude is often used, especially in electrical engineering, to characterize complicated waveforms.
  - Mean amplitude of a repeated movement, i.e., mean displacement range of the averaged segments corresponding to identified time windows (length of 600 ms). Usually this displacement pattern is similar to a photoplethysmography waveform. Kucewicz et al. [19] called this parameter a “pulse amplitude”. During this investigation we use the absolute value of amplitude, meanwhile Kucewicz et al. [19] retained positive and negative values of amplitudes to discern directions of micromovements.
2. Strain we define as a module of the derivative of mean amplitudes of a repeated movement calculated along the ultrasound scanning line’s direction. Therefore, this parameter reflects a level of spatial non-homogeneity of the endogenous strain. Although strain elsewhere can be computed in various ways [14,39,40], a higher strain means higher mean amplitude differences between adjacent spatial points. The strain magnitude was expressed in per-mille (‰), as in the study by Selbekk et al. [14]. Since distances between adjacent spatial points were used as fixed values, our calculated strain is the Lagrangian strain [41,42].
3. Frequency of high-end spectra peak (shortly–FreqHP) was calculated from the entire displacement signal length (6-s). This parameter was chosen as a spectral estimate of displacement waveform morphology. FreqHP was estimated at the peak of the power spectra observed in the frequency range from 1.5 × FreqD to 22.5 Hz with a resolution of 0.1692 Hz. (The higher harmonics present in frequency spectra of cerebral hydrodynamics are observed with various invasive technologies [25,30], however the known approaches of endogenous quasi-periodic displacement US RF detection are all frequency-limited to the bandwidth of the pulse wave [14,18,43]. Thus, here we intentionally look at the spectra high-end frequencies while modifying the method of displacement characterization.)

All the above-mentioned endogenous brain tissue displacement parameters, in various ways, represent quasi-rhythmic non-homogeneous tissue deformation i.e., the complex process both in time and in space. To completely describe this non-stationary process in the context of brain tissue degeneration, statistical distributions of parameters were taken into account. The parameters of brain tissue displacement signals are not normally distributed among spatial points: amplitude parameters usually are right-skewed and similar to ex-Gaussian distribution [44,45]; strain has a distribution similar to an exponential one; morphology parameter (FreqHP) has an irregular distribution. Therefore, the distributions of all parameters of all displacement signals were evaluated by their:

- minimal and maximal values
- median and interquartile range (IQR)
- the most frequent value (statistical mode)
- ex-Gaussian parameters *μ, σ, τ*, also ex-Gaussian distribution mean ($\bar{x}=\mu +\tau$) and standard deviation ($SD=\sqrt{\sigma^{2}+\tau^{2}}$). (Calculated by using the DISTRIB toolbox for MATLAB [44]; the first two parameters (*μ* and *σ*) correspond to the mean and standard deviation of the Gaussian component of ex-Gaussian distribution; parameter *τ* is the mean of the exponential component of ex-Gaussian distribution; a larger *τ* refers a more skewed distribution.)

The variety of simultaneously evaluated sets of parameters and the quantification of individual point distributions in the ROI (rather than stopping at individual points or on an average of points) is the novelty of this study.

### 2.4. Statistical Analysis

Since majority parameter estimates were not normally distributed, they were compared between healthy control subjects and patients with AD using the non-parametric Mann–Whitney test. Variables that achieved a p value of less than 0.25 were examined with multivariate analysis using logistic regression (LR). Forward and backward stepwise LR was applied to assess the predictive characteristics of the three groups of parameters (amplitude, strain, and morphology (FreqHP) parameters) between AD patients and control subjects, with age included as a covariate. 

Receiver operating characteristic (ROC) analysis was used to evaluate the performance of the diagnostic ability of the analyzed parameter estimates and—more generally—to evaluate the accuracy of the LR model that classified subjects into sick or healthy. An optimal diagnostic cut-off point was determined via the Youden index. ROC curves were analyzed to define a cut-off value for the highest sensitivity and specificity of the RF US parameter estimates, which achieved *p* ≤ 0.05 using a Mann–Whitney test, or were included into LR models.

The significance level α was set at 0.05. Statistical analysis was performed using IBM SPSS Statistics 22.0 (IBM Corp., Armonk, NY, USA) software.

## 3. Results

The mean age of AD patients was 71.8 ± 8.8 years, 11 (55%) of them were men. The mean age of the healthy control subjects was 68.0 ± 6.5 years, 10 (50%) of them were men. Thus, groups were similar regarding age and sex (*p* > 0.05). AD patients were less educated (13.0 ± 3.6 years) compared to control subjects (15.2 ± 3.2 years), although a difference was not statistically significant (*p* = 0.051). The mean MMSE score was 17.6 ± 8.8 for AD patients, and 29.2 ± 1.1 for control subjects (*p* < 0.001).

Once the recordings of the US RF data were processed, six subjects from the AD patient group and four subjects from the control group were excluded due to poor quality data (too noisy or not having a sufficiently repeated motion pattern along the scanning line axis). Therefore, US data of 16 control subjects (the ROIs of both sides for five subjects, only right ROI for six subjects, only left ROI for five subjects—21 ROIs in total) and of 14 AD patients (the ROIs of both sides for five patients, only right ROI for six patients, only left ROI for three patients—19 ROIs in total) were used for the final analyses. Age differences between the remaining control subjects (68.5 ± 6.2 years) and AD patients (70.9 ± 9.7 years) was not statistically significant (*p* = 0.4340). Nine persons (56.3%) of the control group were women and six of them were men (42.8%) in the AD group. The MMSE score was significantly lower (18.5 ± 3.6) for AD patients compared to the control subjects (29.2 ± 1.1) (*p* < 0.001). Education of the remaining subjects did not differ statistically between the groups (13.8 ± 3.2 in AD group 15.6 ± 2.3 healthy controls group, *p* = 0.085).

### 3.1. Estimates of Particular Parameters of Displacement

**Figure 1:**
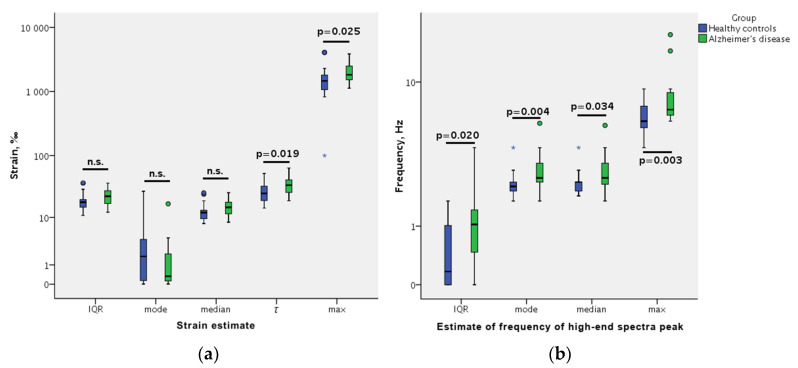
Displacement parameter estimate comparisons between healthy controls and patients with Alzheimer’s disease (AD): (**a**) strain and (**b**) frequency of high-end spectra peak (FreqHP). AD patients had a longer exponential tail of strain distribution (as evaluated by ex-Gaussian distribution *τ* and maximum estimates), FreqHP (as evaluated by mode, median and maximum estimates), and a higher variability of FreqHP (as evaluated by interquartile range (IQR) estimate). Boxplots represent median (midline of box), Q1 and Q3 (lower and upper side of box, respectively); whiskers are no greater than 1.5 × IQR. *P* values are according to the Mann–Whitney test; n.s. = non-significant differences.

Displacement parameters—strain and morphology (FreqHP)—differed significantly between the AD patients and the control subjects according to the Mann–Whitney test: AD patients had a longer exponential tail of strain distribution (as evaluated by ex-Gaussian distribution *τ* and maximum estimates), a higher FreqHP (as evaluated by mode, median and maximum estimates), and a higher variability of FreqHP (as evaluated by IQR estimate) (see Figure 1). However, each single parameter estimate had only a poor or fair area under the curve (AUC) in ROC analysis (see Table 1). Displacement amplitude parameter estimates did not differ between the AD patients and the control subjects. The dominant frequency (FreqD) of the low-end spectrum peak (expected heart rate) did not differ between groups (Mann–Whitney *p* = 0.347).

### 3.2. Models of Logistic Regression Analysis

To assess the predictive power of all types of parameters together, analyzed for the likelihood that the subject had AD, forward and backward stepwise LR was applied and four regression models developed.

**Figure 2:**
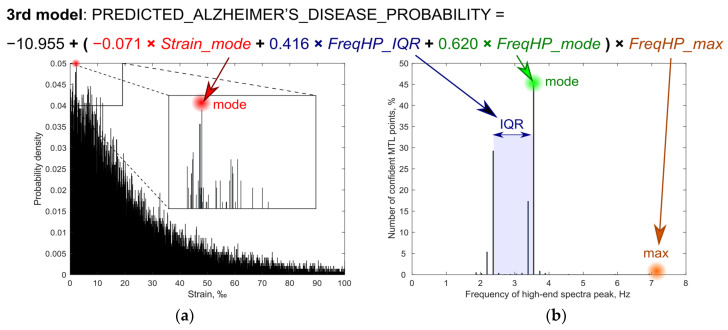
Visualization of four parameter estimates which were included into logistic regression models. Example based on the third model. Sub-images represent: (**a**) distribution of strain and (**b**) frequency of the high-end spectra peak (FreqHP) of confidently repeatable moving individual points, from medial temporal lobe (MTL). FreqHP_IQR—FreqHP interquartile range, Hz; FreqHP_max—FreqHP maximum, Hz; FreqHP_mode—FreqHP mode, Hz; Strain_mode—strain mode, ‰.

All regression models (presented in Table 2) were statistically significant, had a good quality of fit as explained by 79.5–81.0% of the variance in group membership, and correctly classified 95% of cases. An increase in three estimates of the FreqHP (IQR, mode, maximum), and a decrease in strain mode was significantly associated with AD, independent of age. The first two models included variables without their interactions, however their Exp(β) 95% confidence intervals were extremely large. Additionally, forward and backward stepwise LR produced different models: in a forward model just two estimates of FreqHP—maximum and IQR—were left as significant to predict AD. Therefore, despite correlation analysis not revealing any significant correlations between the variables, few cases had a high FreqHP maximum and low other estimates, also—vice versa—few cases had a low FreqHP and high other estimates. Thus, interactions of the variables from the first model were included, and three of them reproduced the same subject classification accuracy of 95% into one of the two groups—AD patients or healthy controls. This time, the model appeared to be optimal. Interactions of FreqHP maximal values with the most frequent FreqHP value and with IQR, as well as an interaction of strain with FreqHP maximal values, were associated with AD independent of age. Thus, the third model is the most favorable—its visualization is provided in Figure 2.

Results of ROC analysis of parameter estimates are presented in Table 1 and ROC analysis of the predicted probability of LR models are in Table 3. Based on ROC curves of LR model predictions, the optimal cut-off values were defined to classify a subject into one of two groups—AD or healthy controls. All models had a cut-off from 61.0 to 68.5% and separated the patients from the healthy subjects with a sensitivity of 89.5% and a specificity of 100%. The AUC of predicted probability in all models was excellent (from 95.2 to 95.7%).

## 4. Discussion

We aimed to assess the diagnostic ability of endogenous brain tissue displacement parameters in the MTL using US RF signals for the differentiation of patients with AD from healthy controls. We demonstrated that the evaluation of complex interactions between the proposed set of brain tissue displacement signal parameters allowed the detecting of the MTL pathology with substantial diagnostic ability. According to our models, AD patients can be differentiated from healthy controls by a sharper morphology of some individual MTL spatial point displacements (i.e., by spreading the spectrum of displacements to the high-end frequencies with higher variability across spatial points within the region), by lower displacement amplitude differences between adjacent spatial points (i.e., lower strain), and by a higher interaction of these attributes.

Notably, the measured/observed endogenous brain tissue displacements depend not only on brain tissue properties per se, but also depend on the dynamics of the cardiovascular system and measurement/calculation methods; these two factors and the lack of similar studies makes our results hardly directly comparable to the findings of other authors.

Brain tissue and cardiovascular changes (or the extent of these changes) caused by age and by AD partly overlap—stiffening of arteries, a decrease of blood perfusion and autoregulatory function—and probably are additional causes of brain tissue damage influencing AD [46,47,48]. Although age was matched between participants, our models remained unchanged even while entering age as a covariate.

This result—an achieved ability to differentiate AD from healthy controls—possibly could be attributed to our method [20] modifications applied for the first time here. Those novel features lie in the applied high-end frequency spectrum parameter for characterization of brain tissue atrophy caused by AD. Other modifications of the proposed method included removing shared movement of an entire ROI to enhance the signal and noise ratio and enhance differences inside the ROI, limiting the analysis to the confidently repeatable moving individual points, complex evaluation of the variety of parameters, and the quantification of the distribution of individual points in an ROI (rather than averaging them).

During our study, no single parameter estimate was sufficiently powerful to discern AD patients from healthy control subjects according to ROC analysis—the simultaneous interaction of several frequency domains (FreqHP) and one spatial domain (strain) parameter estimates was necessary to achieve excellent specificity and a very good (89.5%) sensitivity in our proposed model. Having in mind that the Yilmaz et al., [11] method, based on a single structural parameter estimate (a ratio of the MTL height and the choroidal fissure), achieved an AUC of 0.81 (a sensitivity of 83%, a specificity of 76%) for AD diagnosis, we could hypothesize that—if this single variable would be combined with US RF-based tissue displacement parameters—maybe an even more accurate tool for the diagnosis of AD and follow up with the patient over the disease’s course could be created.

Seen in our model, displacement morphology parameter (FreqHP—distribution of frequency values by finding a peak with the maximum power within high-end spectra for every spatial point separately) estimates have a higher weight for AD diagnostics than tissue strain. Unfortunately, explicit interpretation of this observation in light of previous research cannot be done as, to the best of our knowledge, there are no comparable studies. The most favorable of our models (Third LR model) could be expressed as the multiplication of two components: (1) the sum of the weighted most frequent value of FreqHP distribution, interquartile range of FreqHP distribution and, (with relatively little weight) most frequent strain value; (2) maximal FreqHP value. This form of two components mainly reflects the frequency of micromovements inside MTL and, thus, could be compared to quadratic function expression of AD probability. Goto et al. [49] found a quadratic function relationship between the intracranial pressure value and the pulse waveform frequency inside the brain. Seen in the Goto et al. [49] study, the lowest frequency of pulse waveforms was higher than the frequency values of the high-end spectra in our study. Although we cannot link our results to the Goto et al. [49] findings directly, Goto et al. provide an insight that high frequency also could have a pathophysiological meaning.

The maximal value of FreqHP has the highest weight in our models. These maximal peak frequency values are near to frequency peaks visible by other researchers [22,31] and we can only guess that these frequency peaks presumably could correspond to tidal waves (which correlate to arterial pulses reflected off the brain parenchyma), dicrotic notch [24] and/or microcirculation [48]. However, we need further investigations to explain the meaning of our results regarding micromovement morphology.

Although the maximal strain was higher (i.e., exponential tail of strain distribution was statistically significantly longer) in AD patients, the related strain estimates did not result in our LR models. Meanwhile, the LR models benefited from the most frequent value of strain, which was not statistically significantly different between AD patients and healthy controls: the higher strain mode contributed to a lower probability of AD. This discrepancy of strain estimates, while being preliminary, suggests that the strain mode in our models could act only as a modifier of frequency domain estimates. However investigation of a more precise interaction between variables and inclusion of new pathologies could be an object of future studies. Although strain elsewhere is correlated to tissue elasticity [50], we discourage interpreting our obtained strain values in this way because such interpretation would need data of applied forces (and/or their distribution) and, while comparing strain values from different studies, a compatibility of methodologies should be taken into account. The quantitative methods (including MRI) on the same brain structures still report different stiffness values due to unstandardized experimental protocols [15,51].

The excellent specificity and very good sensitivity of our proposed models cannot be taken as evidence for AD diagnostics, yet. The limitations of this study are a small initial number of participants, a high percentage of excluded data due to unrepeated movement of the brain tissue. Simultaneous electrocardiogram was not recorded; however, it could help to select global time windows or repeated movement windows and to monitor heart rate. The classical 1D cross-correlation that we have used in our study can only access displacements along the scanning lines. Lack of information about blood circulation in the brain, which could be possible using transcranial Doppler technologies, also would be a supplementary source of valuable information making the interpretation of study results even more comprehensive. 

Notwithstanding its limitations, accurate tracking of endogenous brain tissue displacements and complex estimation of its parameter distributions probably reveal some features of complex mechanisms influencing the brain tissue changes specific to AD. This paves a way toward more explicit interpretations of tissue characterization and clinical diagnostic applications. Future studies are needed to validate the results of our exploratory study using larger cohorts of patients and of healthy subjects. Diagnostic accuracy of the method should be confirmed using different pathologic entities of brain diseases. Future studies could investigate more US parameters, especially space-dependent distribution of endogenous movements, simultaneously while seeking a better diagnostic tool, e.g., combine our model with the Yilmaz et al., [11] method and other potential measures. Particular attention could be paid to the analysis of endogenous pulsation sources in the brain structures and differential methods of the quantification of movement parameters.

## 5. Conclusion

AD patients can be differentiated from healthy controls by a sharper morphology of some individual MTL spatial point displacements, by lower displacement amplitude differences between adjacent spatial points, and by a higher interaction of these attributes.

## Figures and Tables

**Table 1:** Receiver operating characteristic (ROC) analysis of variables that achieved *p* ≤ 0.05 using a Mann–Whitney test or were used in logistic regression models.

Parameter Estimate	AUC, %	95% Confidence Interval	Cut-Off	Sensitivity, %	Specificity, %
FreqHP max, Hz	77.2	62.6–91.8	6.26	89.5	61.9
FreqHP mode, Hz	75.9	60.3–91.6	2.45	63.2	81.0
FreqHP median, Hz	69.7	52.7–86.6	2.45	57.9	76.2
FreqHP IQR, Hz	71.9	55.6–88.3	0.76	73.7	66.7
Strain ex-Gaussian τ, ‰	71.7	55.9–87.5	23.22	94.7	42.9
Strain max, ‰	70.7	54.4–87.0	1308.07	94.7	42.9
Strain mode, ‰	61.7	44.2–79.1	1.58	73.7	52.4

AUC—area under a curve; FreqHP_IQR—frequency of high-end spectra peak, interquartile range, Hz; FreqHP_max—frequency of high-end spectra peak, maximum, Hz; FreqHP_mode—frequency of high-end spectra peak, mode, Hz; Strain_mode—strain, mode, ‰.

**Table 2:** The predictive power of medial temporal lobe tissue displacement signal parameters for the likelihood that the subject has Alzheimer’s disease. Results of logistic regression analyses.

Parameter Estimate	β	Exp(β)	Exp(β) 95% Confidence Interval	*p* Value
**1st Model**				
FreqHP_IQR	2.396	10.976	1.021–117.985	0.048
FreqHP_mode	4.599	99.424	3.312–2984.675	0.008
FreqHP_max	1.196	3.306	1.040–10.506	0.043
Strain_mode	−0.510	0.600	0.390–0.926	0.021
Constant	−19.838	<0.001	-	0.002
**2nd Model**				
FreqHP_IQR	2.377	10.776	0.969–119.885	0.053
FreqHP_mode	4.455	86.028	2.744–2696.678	0.011
FreqHP_max	1.144	3.138	1.026–9.597	0.045
Strain_mode	−0.498	0.607	0.392–0.942	0.026
Age	0.040	1.041	0.852–1.272	0.696
Constant	−21.989	<0.001	-	0.013
**3rd Model**				
FreqHP_IQR × FreqHP_max	0.416	1.516	1.025 2.240	0.037
FreqHP_mode × FreqHP_max	0.620	1.859	1.216–2.841	0.004
Strain_mode × FreqHP_max	−0.071	0.931	0.879–0.987	0.016
Constant	−10.955	<0.001	-	0.002
**4th Model**				
FreqHP_IQR × FreqHP_max	0.410	1.507	1.015–2.235	0.042
FreqHP_mode × FreqHP_max	0.600	1.821	1.181–2.809	0.007
Strain_mode × FreqHP_max	−0.069	0.933	0.880–0.989	0.020
Age	0.027	1.028	0.841–1.255	0.789
Constant	−12.573	<0.001	-	0.082

FreqHP_IQR—frequency of high-end spectra peak, interquartile range, Hz; FreqHP_max—frequency of high-end spectra peak, maximum, Hz; FreqHP_mode—frequency of high-end spectra peak, mode, Hz; Strain_mode—strain, mode, ‰.

**Table 3:** ROC analysis of the predicted probability of logistic regression models for the likelihood that the subject has Alzheimer’s disease.

Predicted Probability in Model	AUC, %	*p* Value	95% Confidence Interval	Cut-Off, %	Sensitivity, %	Specificity, %
1st model	95.5	<0.001	88.6–100.0	60.98	89.5	100.0
2nd model	95.2	<0.001	87.9–100.0	66.53	89.5	100.0
3rd model	95.5	<0.001	88.3–100.0	65.20	89.5	100.0
4th model	95.7	<0.001	88.7–100.0	68.47	89.5	100.0

## Abbreviations

AD	Alzheimer’s disease
AUC	area under a curve
CSF	cerebrospinal fluid
IQR	interquartile range
FreqD	dominant frequency
FreqHP	frequency of high-end spectra peak
LR	logistic regression
MMSE	Mini-mental state examination
MRI	magnetic resonance imaging
MTL	medial temporal lobe
PET	positron emission tomography
RF	radiofrequency
ROI	region of interest
ROC	receiver operating characteristic
RMS	root mean square
TCS	transcranial sonography
US	ultrasound
